# Time-Resolved Characterization of Indoor Air Quality due to Human Activity and Likely Outdoor Sources during Early Evening Secondary School Wrestling Matches

**DOI:** 10.1155/2021/5580616

**Published:** 2021-06-07

**Authors:** Derek G. Shendell, Lauren N. Gonzalez, Joseph A. Panchella, Jason Morrell

**Affiliations:** ^1^Rutgers School of Public Health (SPH), Department of Environmental and Occupational Health and Justice, Piscataway, NJ, USA; ^2^NJ Safe Schools Program, Rutgers SPH, Piscataway, NJ, USA; ^3^West Deptford School District, West Deptford, NJ, USA

## Abstract

Despite positive health outcomes associated with physical activity as well as individual and team sports, poor indoor air and environmental quality can adversely affect human health, performance, and comfort. We conducted a 14-month field case study incorporating two winter sports regular seasons (12/2017–2/2019) including analyses of particulate matter (PMx) in air and in dust, carbon dioxide (CO_2_), temperature, and relative humidity (RH%) during secondary or high school wrestling activities in southern New Jersey, USA. After planning and piloting methods during the first winter sports regular season (12/2017–2/2018), during the second winter sports regular season (1–2/2019), we conducted a purposeful simultaneous real-time sampling midgymnasium adjacent to the wrestling mats. Gymnasium occupancy ranged 100–500 people. Data collected included inhalable PM_10_ resuspended from floor mats, fine respirable PM_2.5_, and indoor CO_2_, temperature, and RH%. Short-term real-time elevated PM_10_ levels were directly compared with simultaneously documented wrestling match bouts, e.g., student-athlete takedowns and pins. PM_10_ and PM_2.5_ levels were compared with other known documented activities indoors (e.g., snack bar) and sources outdoors (e.g., adjacent parking lot and major freeway). To understand CO_2_, temperature, and RH% data, we characterized the HS gym mechanical ventilation system—no doors or windows outdoors—and recorded occupancy during match hours. Indoor CO_2_ levels ranged ∼700–1000 ppm during match #1 on 1/23/2019 but ranged from ∼900 to 1900 ppm during match #2 on 1/30/2019, with >1000 ppm for the majority of the time (and throughout the entire varsity match when occupancy was at maximum). Future research should further characterize PM_10_ constituents in mat dust and indoor air with larger samples of schools and matches.

## 1. Introduction

Children are more vulnerable to poor indoor air and environmental quality in schools [1–5]. Indoor air quality (IAQ) or indoor air and environmental quality in schools have been extensively studied, with a focus on classrooms (e.g., Heudorf et al.) [1] or university sports facilities, including those used for gymnastics [2].

When exercising, the velocity of airflow increases, meaning particulate matter (PM) present in the air can penetrate deeper into the respiratory tract [6]. Levels of airborne PM in commercial and university gyms have been quantified by groups based in Europe [2, 7]. School gymnasiums in Prague, Czech Republic, [8] and Cassino, Italy [9], have been studied during normal use, but specific time-course studies examining PM levels during specific sports activities in secondary schools are rare.

Poor IAQ has been repeatedly associated with adverse effects on human health and performance and thermal comfort. Temperature (T), relative humidity (RH%), and carbon dioxide (CO_2_) contribute to IAQ. Increased respiration during exercise signifies quicker, deeper breaths in and increased CO_2_ expelled; individual energy use relates to CO_2_ generated through metabolic activity [9]. Previous research is limited on the effects of inadequate ventilation on the human system while exercising [6, 9]. However, previous studies on school gyms and commercial offices suggested an inefficient mechanical HVAC (heating, ventilation, and air conditioning) system can lead to elevated indoor CO_2_ concentrations—above or near the higher end of “normal” (350–2500 parts per million or ppm) [10]. Elevated indoor CO_2_ concentrations can then affect performance and impact the health of a building's occupants, e.g., symptoms of sick building syndrome [10]. A guideline for indoor CO_2_ (nonoccupational setting) is 1000 ppm [11]; 5000 ppm CO_2_ is the US occupational enforceable regulatory limit [12]. There are also guidelines for *T* and RH% as part of assessments of indoor occupant thermal comfort across seasons of the year [13].

Our objective was to conduct a study over two consecutive winter sports seasons (with pilot sampling completed in the first winter sports season [14, 15]) to determine PM levels during wresting practices and within individual bouts, team matches, and multiteam meets hosted by one secondary or high school (HS). The student-athletes in this study competed for multiple southern New Jersey (NJ) public HS. This paper summarizes key results from the three components of the second winter sports season [16–18] of our study. First, we focused on variability in indoor air inhalable PM_10_, as influenced by dust resuspension from surfaces (gym floor, wrestling mat) due to human activity. Second, we also focused on indoor fine, respirable PM_2.5_, potentially also influenced by outdoor PM_2.5_ generated by a major freeway near the HS (next to entry road and parking lot). Finally, we assessed IAQ beyond air and dust (floors, mats) related to ventilation—mechanical HVAC system without natural ventilation via open doors or windows to the outdoors—with measures of CO_2_, *T*, and RH%.

## 2. Materials and Methods

This was a field research case study in the 2^nd^ regular HS wrestling season in winter late 2018-early 2019, after planning was conducted and methods were piloted in the 1^st^ regular HS wrestling season in winter late 2017-early 2018. Thus, the overall study period was 14 months. Regular season schedules in HS wrestling in the State of New Jersey dictate each HS may only host a few meets in the winter sports season each school year at their HS. Sampling was conducted at a southern New Jersey public secondary school, School A, on consecutive weeks January-February 2019. Other local public secondary schools, Schools B-D, also participated in matches during the regular season matches at School A. Matches took place on two separate days during late afternoon-evening times, approximately 2-3 hours. There were weekly HS regular season (nontournament) wrestling matches in three home meets between ≥ 2 teams hosted at School A. The first match on 1/23/2019 was between two schools and had about 100–150 attendees (including spectators, student-athletes, and coaches), and the second match on 1/30/2019 was between two schools and had about 450–500 spectators. The School A gym had no operable windows to the outdoors, had ceiling lighting, and had no doors directly to the outdoors. The doors to this gym, which were closed during matches/bouts or only opened periodically for spectator entrance or exit, were connected to hallways serving interior offices and classrooms/meeting rooms. This gym had a mechanical HVAC system located above it on the roof. A thorough assessment of the exact operational details of this HVAC system was beyond this study's scope. This gym's dimensions are ∼100 feet long (∼30.5 meters), ∼80 feet wide (∼24.4 meters), and ∼25 feet high (∼7.6 meters). The gym's maximum capacity, based on state and local fire code limits, is 1200 people standing (with/without retractable bleachers used), 1000 with chairs, or 500 with chairs and tables.

**Figure 1:**
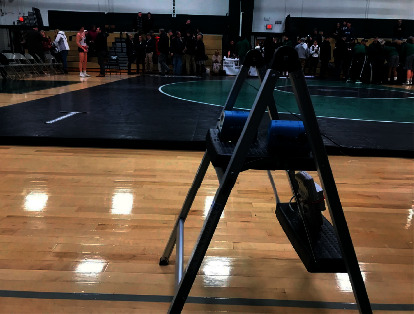
Placement of IAQ samplers inside the participating HS gymnasium near wrestling mats (photo courtesy of L.N. Gonzalez, January 2019).

The location of IAQ sampling equipment used is shown in Figure 1. The *T*, RH, and CO_2_ sensors were ∼0.5 m above the gym floor; blue samplers were particle (PM_2.5_ and PM_10_) counters, ∼1 m above the floor. It is a typical field research practice to locate particle counters at least ∼1 m from floors, walls, and ceilings. It was appropriate to have the sampler with sensors for *T*, RH, and CO_2_ at ∼0.5 m [13] given the focus on wrestling competition. These student-athletes are typically crouched down or on the mat, not standing straight up, while in action. Moreover, this was as close as coaches and officials would allow samplers to be placed, given our concerns for safety and to not disturb, disrupt, or intrude the student-athletes.

### 2.1. Particulate Matter (PM_10_ and PM_2.5_)

Two AM510 SidePak Personal Aerosol Monitors (TSI Incorporated, Shoreview, MN, USA) [19, 20] were used to monitor PM, one for PM10 and another for PM2.5, continuously. These samplers were described in more detail and successfully used in other prior field studies we have conducted [21, 22]. During matches (see Figure 1), both samplers were placed midgymnasium adjacent to the mat and measured PM levels once per second. Activities and significant events performed by the student-athletes during the bouts/matches, including pins and takedowns, were recorded by the study team field technician in a field notebook with a time/date stamp. The watches had been synchronized to the samplers during preparations and calibration at the study team's field dry lab at Rutgers University.

### 2.2. Carbon Dioxide (CO_2_), Temperature (*T*), and Relative Humidity (RH%)

Two Telaire 7001 direct reading monitors internally logged CO_2_ data inside the gym and simultaneously recorded CO_2_ data onto HOBO U12 Data Loggers (Onset Corp, Bourne, MA)—also collecting *T* and RH% data—connected by CO_2_ monitor adapter cables. Samplers were calibrated with compressed nitrogen gas (0 ppm air) before and after each field site visit. Telaire and HOBO sampler logging intervals were programmed as 10-second averages of ten, 1-second data to maximize data storage. Indoors, i.e., inside the gym (see Figure 1), the Telaire 7001 and HOBO U12 combination of sensors and loggers were carefully collocated with the two AM510 monitors (Figure 1). We ensured the two AM510 exhaust air pointed away from the air intakes of each other as well as the Telaire 7001.

### 2.3. Other Data Collected

Separately, outdoor *T* and RH% were recorded during the outdoor PM_2.5_ sampling in a parking space on a small stand on top of a parked car roof. The parking space was adjacent to the gym's building. PM_2.5_ and CO_2_ data are not reported in detail in this paper, given extreme outdoor conditions—cold (range <20 to about 10°F, low RH%)—led to disruptions in sensor-to-data logger performance. In summary, background outdoor CO_2_ did not vary in this study due to relatively lower evening traffic volumes on both an adjacent primary road serving the HS parking lot entrance and exit areas and a freeway <100 meters away from the HS gymnasium. We wanted to confirm, in this paper, indoor CO_2_, not indoor minus outdoor CO_2_, remained the focus because prior research had documented how ambient CO_2_ may vary with distance from major roadways and freeways in areas without other major industrial combustion sources beyond mobile line sources. [21].

### 2.4. Data Management and Analyses

Sampler data were uploaded from each monitor and logger into Microsoft Excel and broken down by time and activity type, during practice, or each match. Descriptive statistics (range or minimum and maximum presented in this paper) and graphs were generated.

## 3. Results and Discussion

### 3.1. PM_10_

**Figure 2:**
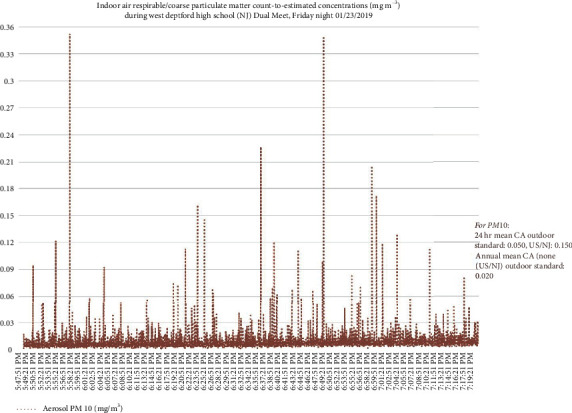
Respirable or coarse particulate matter (PM_10_) count-to-estimated concentrations, mg m^−3^, for match #1 on 1/23/2019. Each team had ∼25 student-athletes and ∼5 coaches/staff (including the certified athletic trainers) present. Notes: values to the right of peaks above were the highest measured values (off *y*-axis). Varsity warm-up on mats started ∼17 : 50, then match (individual bouts) started ∼18 : 03 and ended ∼19 : 11; student-athletes and coaches completed clean-up/roll mats to ∼19 : 20.

**Figure 3:**
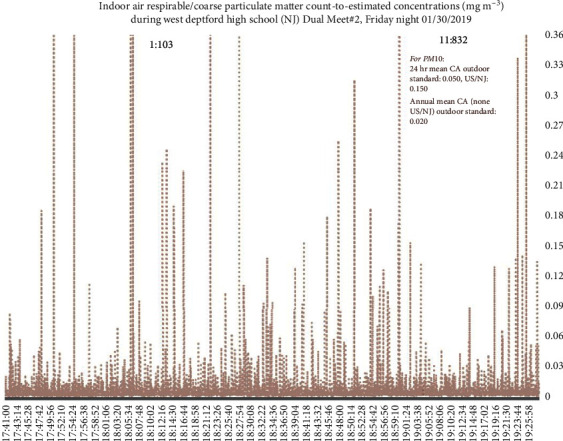
Respirable or coarse particulate matter (PM_10_) count-to-estimated concentrations, mg m^−3^, for match #2 on 1/30/2019. Each team had ∼25 student-athletes and ∼5 coaches/staff (including the certified athletic trainers) present. Notes: values to the right of peaks above were the highest measured values (off *y*-axis). Junior varsity match finished ∼17 : 40, varsity warm-up exercises on mats to ∼18 : 00, then match (individual bouts) started ∼18 : 00 and ended ∼19 : 24, and student-athletes and coaches clean-up/roll mats ∼19 : 24–19 : 29 (data loggers stopped by 19 : 30 on study days).

**Figure 4:**
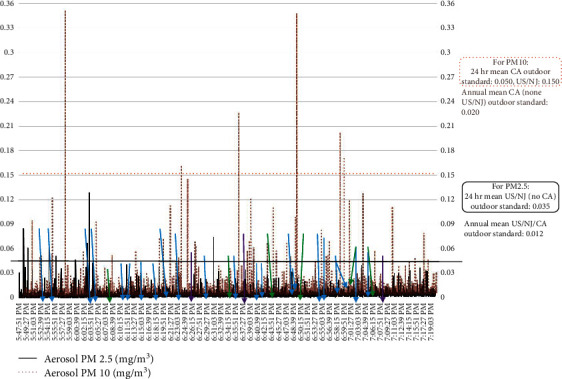
Respirable or coarse particulate matter (PM_10_) and fine particulate matter (PM_2.5_) count-to-estimated concentrations, mg m^−3^, and suggested impact of student-athlete activities, match #1, 1/23/2019. Each team had ∼25 student-athletes and ∼5 coaches/staff (including the certified athletic trainers) present. Notes: the blue arrows were takedown maneuvers; the green arrows were pins; and the purple arrows were when a takedown maneuver led to a pin. For 1/23/2019, varsity warm-up on mats started ∼17 : 50, then match (individual bouts) started ∼18 : 03 and ended ∼19 : 11; student-athletes and coaches completed clean-up/mats rolled to ∼19 : 20 (data loggers stopped by 19 : 30 on study days).

**Figure 5:**
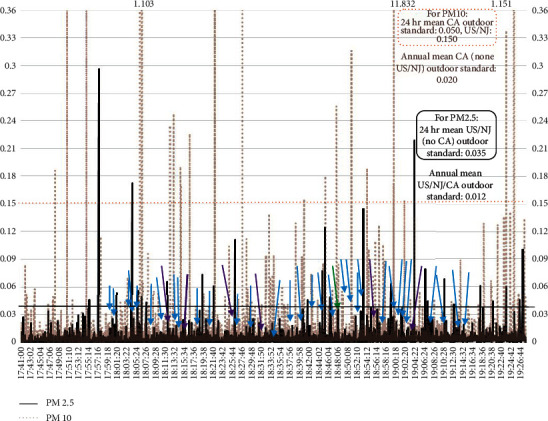
Respirable or coarse particulate matter (PM_10_) and fine particulate matter (PM_2.5_) count-to-estimated concentrations, mg m^−3^, and suggested impact of student-athlete activities, match #2, 1/30/2019. Each team had ∼25 student-athletes and ∼5 coaches/staff (including certified athletic trainers) present. Notes: values to the right of the peaks above were the highest measured values (off *y*-axis). The blue arrows were takedown maneuvers; the green arrows were pins; and the purple arrows were when a takedown maneuver led to a pin. For 1/30/2019, junior varsity match finished ∼17 : 40, varsity warm-up exercises on mats to ∼18 : 00, then match (individual bouts) started ∼18 : 00 and ended ∼19 : 24, and student-athletes and coaches clean-up/mats rolled ∼19 : 24–19 : 29 (data loggers stopped by 19 : 30 on study days).

Figures 2–5 present examples of time series plots on different study days to illustrate how specific activities such as takedown or pinning maneuvers on wrestling mats, or times when many student-athletes were on the mat, such as warm-up exercises and cleaning up/rolling the mats, generated more indoor air PM_10_ in participant breathing zones. Furthermore, in Figure 3, PM_10_ levels are frequently higher compared to Figure 2. This may be partially attributed to more spectators on 1/30/2019 (∼450 individuals present, range ∼400–500) versus on 1/23/2019 (∼100 individuals present).

### 3.2. PM_2.5_

**Figure 6:**
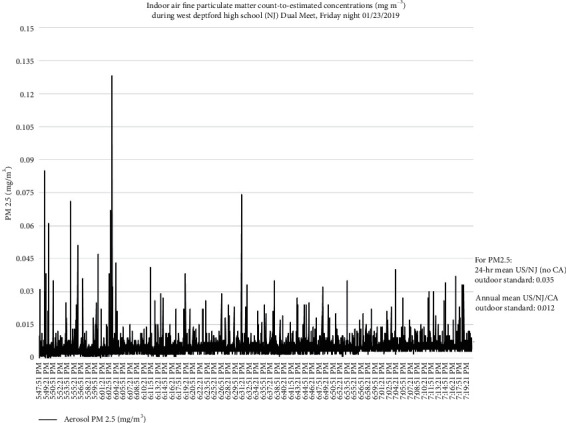
Fine particulate matter (PM_2.5_) count-to-estimated concentrations, mg m^−3^, for match #1 on 1/23/2019. Each team had about 25 student-athletes and five coaches/staff (including certified athletic trainers) present. Notes: varsity warm-up on mats started ∼17 : 50, then the match (individual bouts) started ∼18 : 03 and ended ∼19 : 11; student-athletes and coaches completed clean-up/mats rolled to ∼19 : 20.

**Figure 7:**
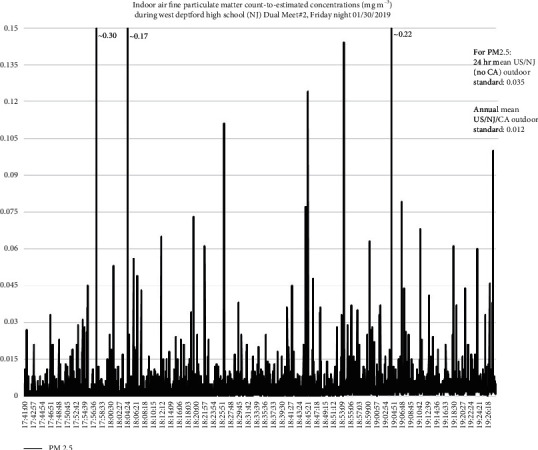
Fine particulate matter (PM_2.5_) count-to-estimated concentrations, mg m^−3^, for match #2 on 1/30/2019. Each team had about 25 student-athletes and five coaches/staff (including certified athletic trainers) present. Notes: junior varsity match finished at ∼17 : 40, varsity warm-up exercises on mats to ∼18 : 00, then match (individual bouts) started ∼18 : 00 and ended ∼19 : 24, clean-up/mats rolled to ∼19 : 24–19 : 29 (data loggers stopped by 19 : 30).

Figures 4–7 present examples of time series plots on different study days to illustrate how specific activities influence indoor air PM_2.5_ in participant breathing zones on the wrestling mats. Specific activities can be speculated, such as warm-up exercises and takedown or pinning maneuvers, within each bout generated variable smaller peak measures, whereas larger peak measures may be influenced by outdoor sources such as vehicles idling, passing, or parking near the gym at the beginning of each match time as well as relatively higher traffic volume on a freeway (US Interstate 295) near the HS gym area. This is also consistent with a recent literature review paper about human exposure to air pollution in indoor sports facilities like gymnasiums and sports halls serving communities [23].

### 3.3. CO_2_, *T*, and RH%

We collected data on different study days in January 2019 to illustrate how occupancy and specific activities on wrestling mats generated more indoor CO_2_ within participant breathing zones. The CO_2_ data were in parts per million, ppm, as an indicator of mechanical ventilation and of occupancy. Indoor CO_2_ levels ranged ∼700–1000 ppm on 1/23/2019 but ranged from ∼900 to 1900 ppm on 1/30/2019, with >1000 ppm for the majority of time (and throughout the entire varsity match). The CO_2_ data were simultaneously compared to concurrent indoor *T* (degrees Celsius, °C; range on both study days ∼18–23°C) and indoor RH (percent, %; range ∼30–35% on 1/23/2019 and ∼25–31% on 1/30/2019).

We also examined initial raw data time series plots of study days (refer to an example, merging 1/23/2019 and 1/30/2019 data, provided in the Supplemental Figure). Data suggested not only specific activities on wrestling mats—warm-up activities/exercises, maneuvers, roll-up mats—but also the increased occupancy during the match on 1/30/20219 compared to that on 1/23/2019 generating greater variability in and more indoor CO_2_ within participant breathing zones. Data were collected on 1/23/2019 while ∼100 spectators were present, and then data were collected on 1/30/2019 with first ∼100 spectators for the duration of the junior varsity match, then ∼400–500 during the duration of the varsity match. Meets were several hours after the end of the school day. Besides the aforementioned spectators, each team had ∼25 people and ∼5 coaches/staff (like the certified athletic trainer).

In summary, specific activities like takedown or pinning maneuvers within a bout combined with inadequate mechanical ventilation for the gym's occupancy during matches led to higher indoor CO_2_ measures. Measured indoor CO_2_ concentrations in this study did not exceed the typical guideline of 1000 ppm [11] except during the 1/30/2019 varsity match when occupancy was higher than typical due to increased numbers of spectators. Also, neither thermal comfort [13] (winter heating season indoor *T* of ∼68–75°F or ∼20–24°C and RH of 20–60% given cold, dry outdoor air) nor fire code occupancy limits were exceeded, and, as schools are workplaces for adults, the measured indoor CO_2_ was below the occupational enforceable regulatory limit [12]. This is consistent with another recent school-based study in Italy [24]; although focused on dominant combustion sources of pollution, researchers also reported differences in concentrations of PM_2.5_ and various chemical pollutants including CO_2_ during times of student activities versus unoccupied, activity-free times. In this study, we could not completely characterize the potential contribution to indoor PM of outdoor PM from the adjacent primary road and a freeway (US Interstate I-295) near School A; however, any other point sources (industry, power plant) were located several kilometers away. Furthermore, gym occupancy relative to its capacity influences CO_2_ levels; during the 1/23/2019 match bouts, about 100 spectators and athletes were present in the gym versus the 1/30/2019 match bouts with up to about 400–500 spectators and athletes during the varsity match. The mechanical HVAC system may not have been set or reset for the increased occupancy expected or realized, respectively, i.e., not enough outdoor air with a lower CO_2_ concentration was brought into the gym. Besides adult and youth spectators present, each team had ∼25 people (student-athletes) and ∼5 coaches and staff (like an athletic trainer) during each meet.

## 4. Conclusions

Young student-athletes are affected by poor IAQ. Airborne particulate matter levels measured near student-athlete breathing zones during athletic events like wrestling on mats can vary widely due to specific activities like takedowns and pins. Many schools have older mechanical HVAC equipment and athletic facilities like gymnasiums may be densely occupied for specific after-school hours during matches or tournaments. This activity can contribute to elevated, variable indoor particle levels; PM_10_ and PM_2.5_ may also be influenced by adjacent outdoor sources if there are no indoor combustion sources. While we could not assess and further test the rooftop HVAC and filters, this study suggested how school mechanical HVAC systems must not only be maintained regularly but also should be set/reset as needed for special events indoors like athletic competitions in the gymnasium to account for known or estimated occupancy, even when stated fire code limits are already consistently followed.

This comprehensive case study warrants more research on IAQ in primary and secondary school gymnasiums and multipurpose rooms used for physical activity/physical education and any interscholastic, club, or intramural sports like wrestling. It can be noted that additional research is even more important in the aftermath of the COVID-19 pandemic 2020–2021 [25, 26]. This worldwide pandemic, caused by the SARS-CoV-2 virus spreading via droplets and aerosols, spanned two school years. Worldwide, the importance of adequate mechanical ventilation with enhanced particle filtration and appropriate sizing for the size of the indoor space as well as the expected daily and maximum occupancy levels—normal or reduced due to the pandemic—was consistently noted across society.

## Data Availability

This study's data are secured on computers per IRB approved stewardship of the NJ Safe Schools Program at Rutgers School of Public Health. Datasets used and analyzed during the current study are available from the corresponding author on reasonable request.

## Supplementary Materials

Supplementary Materials Supplemental Figure: an example of initial raw real-time data output from the Telaire 7100 sensors and HOBO U12 data logger into the HOBO Onset Box Car software as time series plots. Indoor air T (orange, primary *y*-axis), RH% (blue, primary *y*-axis as %), and CO_2_ (grey, secondary y-axis, parts per million or ppm, indicator of mechanical ventilation and of occupancy). Notes: data (to the left of the red vertical line) were collected on 1/23/2019 (∼100 spectators present), and other data (to the right of the red vertical line) were collected on 1/30/2019 (∼100 spectators for the duration of the junior varsity match, then ∼400–500 during the duration of the varsity match). Meets were several hours after the end of the school day. Besides spectators, each team had ∼25 people and ∼5 coaches/staff (like athletic trainer).
