# Supplementary material for: Time-Resolved Characterization of Indoor Air Quality due to Human Activity and Likely Outdoor Sources during Early Evening Secondary School Wrestling Matches
Source: J Environ Public Health. 2021 Jun 7;2021:5580616. doi: 10.1155/2021/5580616 (PMC8205592; doi:10.1155/2021/5580616)
Supplement: Supplementary Materials — Supplemental Figure: an example of initial raw real-time data output from the Telaire 7100 sensors and HOBO U12 data logger into the HOBO Onset Box Car software as time series plots. Indoor air T (orange, primary y-axis), RH% (blue, primary y-axis as %), and CO2 (grey, secondary y-axis, parts per million or ppm, indicator of mechanical ventilation and of occupancy). Notes: data (to the left of the red vertical line) were collected on 1/23/2019 (∼100 spectators present), and other data (to the right of the red vertical line) were collected on 1/30/2019 (∼100 spectators for the duration of the junior varsity match, then ∼400–500 during the duration of the varsity match). Meets were several hours after the end of the school day. Besides spectators, each team had ∼25 people and ∼5 coaches/staff (like athletic trainer). [file 5580616.f1.docx]

**Supplemental Figure.**

An example of initial raw real-time data output from the Telaire 7100 sensors and HOBO U12 data logger into the HOBO Onset Box Car software as time series plots. Indoor air T (orange, primary y-axis), RH% (blue, primary y-axis as %) and CO_2_ (grey, secondary y-axis, parts per million or ppm, indicator of mechanical ventilation and of occupancy). Notes: Data (to left of red vertical line) were collected 1/23/2019 (~100 spectators present), and other data (to the right of the red vertical line) were collected 1/30/2019 (~100 spectators for the duration of the junior varsity match, then ~400-500 during the duration of the varsity match). Meets were several hours after the end of school day. Besides spectators, each team had ~25 people and ~5 coaches/staff (like athletic trainer).
